# Postpartum Women’s Perspectives of Engaging with a Dietitian and Exercise Physiologist via Video Consultations for Weight Management: A Qualitative Evaluation

**DOI:** 10.3390/healthcare6010008

**Published:** 2018-01-19

**Authors:** Lisa Vincze, Megan E. Rollo, Melinda J. Hutchesson, Robin Callister, Debbe I. Thompson, Clare E. Collins

**Affiliations:** 1 School of Health Sciences, Faculty of Health and Medicine, University of Newcastle, Callaghan, NSW 2308, Australia; lisa.vincze@newcastle.edu.au (L.V.); Megan.rollo@newcastle.edu.au (M.E.R.); Melinda.hutchesson@newcastle.edu.au (M.J.H.); 2 Priority Research Centre for Physical Activity and Nutrition, University of Newcastle, Callaghan, NSW 2308, Australia; Robin.Callister@newcastle.edu.au; 3 School of Biomedical Sciences and Pharmacy, Faculty of Health and Medicine, University of Newcastle, Callaghan, NSW 2308, Australia; 4 USDA/ARS Children’s Nutrition Research Center, Department of Pediatrics, Baylor College of Medicine, Houston, TX 77030, USA; Deborah.Thompson@ARS.USDA.GOV

**Keywords:** dietitian, exercise, nutrition, postpartum, telehealth, video consultation, weight

## Abstract

Optimising weight status after childbirth is important. Video consultations are an unexplored opportunity to deliver real-time support to postpartum women to improve lifestyle behaviours. This study aims to provide insight into postpartum women’s perspectives of engaging with a dietitian and exercise physiologist through video consultations for tailored nutrition and exercise care. A qualitative study using individual telephone interviews (13–36 min) was undertaken. 21 women (body mass index (BMI): 28.1 ± 3.8 kg/m^2^; age: 32.3 ± 3.0 years; parity: 1.6 ± 0.9 children) who had completed the 8 week “Video-coaching to assist lifestyle (VITAL) change for mums” intervention participation included up to five video consultations with a dietitian and exercise physiologist. The interviews were audiorecorded and transcribed. Thematic data analysis was conducted by an independent researcher using NVIVO11. Themes relating to the video consultation experience included feeling that they did not differ from other consultations, they were convenient, and the length of time and flexible options were appropriate; however there was a desire for increased contact frequency. The dietitian and exercise physiologist were perceived to increase the participants’ knowledge and confidence to improve health behaviours. The approach to setting realistic and tailored goals was well received. Tailored advice from a dietitian and exercise physiologist received via video consultations is acceptable for postpartum women and offers a viable alternative to in-person care.

## 1. Introduction

Supporting women to achieve a healthy weight in the first year following childbirth has important implications for their life-long weight trajectory, chronic disease risk and offspring health. A recent UK study of 2559 women found that almost three-quarters (73%) retained some gestational weight gain at 6 months postpartum (mean: 3.5 ± 6.2 kg), termed postpartum weight retention (PPWR) [1]. Similar results have been reported in a sample of 152 Australian women, for which 77.5% remained above their pre-pregnancy weight at 6 (median: 4.3 (2.0–8.0) kg) and 67.5% at 12 (median: 4.5 (2.1–8.9) kg) months postpartum [2]. Schauberger reported that women who did not return to their pre-pregnancy weight by 6 months were 8.3 kg heavier 10 years later, compared to women who had returned to their pre-pregnancy weight, who were only 2.4 kg heavier (*p* = 0.01) [3]. Intervening to support the attainment of a healthy weight postpartum and/or a return to pre-pregnancy weight has been the focus of a number of lifestyle interventions for this population subgroup [4,5,6,7]. 

Interventions aimed at limiting PPWR and supporting healthy weight attainment after childbirth have focused on improving nutrition and physical activity behaviours. However, despite women being motivated to make appropriate lifestyle changes [8], success in limiting PPWR has been modest and variable [4]. Elliott-Sale, Barnett and Sale reported that exercise-only interventions had no significant effect on postpartum weight loss (Weighted mean difference (WMD): −1.74 kg; Confidence Interval (CI): −3.59 to 0.10) [9]. Amorim and Linne found that diet-only (Mean difference (MD): −1.70 kg; CI: −2.08 to −1.32) and diet-plus-exercise (MD: −1.93 kg; CI: −2.96 to −0.89) interventions achieved significant weight loss compared to controls [10]. Compromised engagement with lifestyle interventions may be due to the barriers faced by women at this life-stage (i.e., caring and family commitments, and lack of time, knowledge and support) [8,11]. Innovative healthcare approaches are required to engage women at this challenging and complex life-stage and to support them to overcome barriers and make sustainable lifestyle changes in order to successfully limit PPWR.

Video conferencing enables the remote delivery of one-on-one healthcare via real-time audio and video streaming, reducing the need to travel to in-person consultations [12]. With at least 86% of Australian adults having access to the internet [13], providing online real-time support is a viable, yet unexplored opportunity in postpartum healthcare. Nutrition and physical activity care delivered remotely may improve accessibility and convenience and help to overcome many of the barriers impacting engagement with lifestyle changes at this life-stage (i.e., time, and access to childcare and support). Despite a number of interventions targeting women following childbirth, none have yet reported utilising video conferencing to provide one-on-one support for the adoption of healthy lifestyle behaviours [4]. The aim of the “Video-coaching to assist lifestyle (VITAL) change for mums” study was to evaluate, in women who were 3–12 months postpartum, the implementation, acceptability and preliminary efficacy of a personally tailored nutrition and exercise program, delivered via video consultations by accredited practitioners (Accredited Practising Dietitian and Accredited Exercise Physiologist). The aim of the current report is to provide qualitative insight into women’s experiences of participating in VITAL change for mums, specifically their perspectives of engaging with the dietitian and exercise physiologist through video consultations for tailored nutrition and exercise care.

## 2. Materials and Methods

The conduct and reporting of this research adhered to the guidelines outlined in the consolidated criteria for reporting qualitative research (COREQ) [14]. This study was approved by the University of Newcastle Human Research Ethics Committee (H-2015-0369), and the women provided informed written consent prior to completing the telephone interview.

### 2.1. Study Design

Women were interviewed about their experiences of participating in the VITAL change for mums nutrition and exercise intervention for postpartum women. A qualitative design using digitally recorded individual telephone interview data was used. Telephone interviews were utilised because of their potential to facilitate an open and honest dialogue with participants about their experiences in a relatively “anonymous” environment, to try to minimise perceived social pressure and respondent desirability bias [15,16].

### 2.2. Participants and Intervention

Thirty women who were 3–12 months postpartum with a body mass index (BMI) of ≥25 kg/m^2^ or a >2 kg weight retention from their most recent childbirth and who had participated in the 8 week single-arm pre–post VITAL feasibility study were eligible. Detailed methods and outcomes have been reported elsewhere [4]. Briefly, the women were recruited using online methods consisting of social media posts that advertised the study and eligibility criteria. The participants completed baseline (pre-) and follow-up (post-) measurement sessions immediately prior to, and following, the 8 week intervention. The intervention participation included the following: (1) up to five individual real-time video consultations, consisting of two consultations with an Accredited Practising Dietitian, two with an Accredited Exercise Physiologist and one optional self-selected session with either practitioner; (2) the provision of equipment to enable the conduct of video consultations, including an iPad mini, a Gymstick resistance band and a Jawbone UP physical activity tracker; (3) the completion of the Australian Eating Survey (AES) Food Frequency Questionnaire (FFQ) and a 3 day image-based food record captured by the participants and uploaded to Evernote, and subsequent tailored nutrition counselling on the basis of the participant responses and the personalised report generated from the AES FFQ; and (4) two-way utilisation of the file-sharing app Evernote. Video consultations focused on tailored personalised nutrition and exercise advice to support the attainment of a healthy weight following childbirth. All 27 women who completed the VITAL intervention were invited to participate in a telephone interview following the completion of their follow-up assessment. 

### 2.3. Data Collection

All interviews were conducted by a female research team member who was an Accredited Practising Dietitian with PhD qualifications and had no established relationship with the participants. The interviews lasted between 13 and 36 min and were conducted a mean of 28.4 ± 14.3 days following the intervention completion (range: 13–52 days). The interviewer received training with regard to the expected procedure and the conduct of the telephone interviews from the lead author (L.V.). 

The semi-structured interview framework was developed by the research team to facilitate discussion and reflection around women’s experiences of participating in VITAL change for mums. Guiding interview questions were developed in collaboration with a highly experienced qualitative researcher (D.I.T.). Specifically, the areas of enquiry explored were the following: (1) motivations and expectations for participation; (2) participants’ experience with the mode of delivery; (3) perceptions of the components of the VITAL change for mums study. The interview protocol is included as a Supplementary Materials file.

### 2.4. Data Analysis

The interviews were digitally recorded and transcribed verbatim, together with field notes. A computer program (NVIVO 11, QSR International, Melbourne, Australia) was used to assist with the organisational aspects of data analysis. All qualitative analyses were conducted by an independent qualitative researcher. 

A systematic deductive approach to thematic data analysis was adopted, allowing for the identification and refinement of the key concepts that had been developed as a part of the study framework. Initially, a coding framework, firmly grounded in the overall study aims, was formulated. This framework facilitated the development of a taxonomy that would enable a detailed description of the domains that characterised the multifaceted experiences of the program participants, as well as the establishment of relationships within the data. A final coding scheme, developed from the refinement and expansion after the coding of transcripts, was used to code the complete dataset. Themes and relationships were identified and described in detail. All themes are presented in weighted order with the most frequently mentioned first. 

## 3. Results

**Figure 1:**
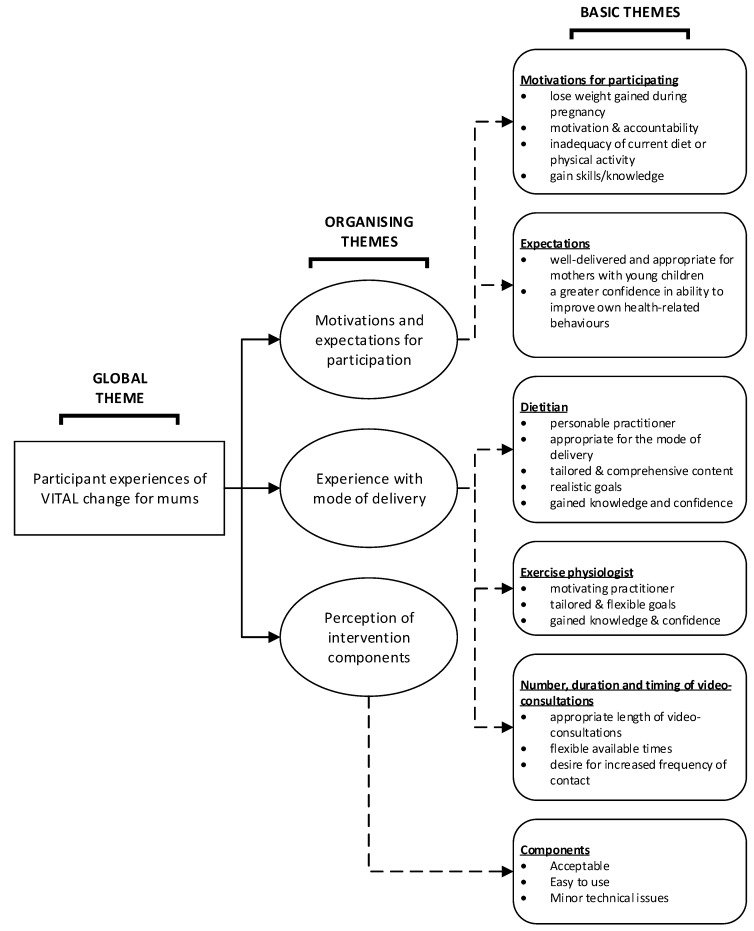
Thematic network of postpartum women’s experiences of participating in Video-coaching to assist lifestyle (VITAL) change for mums.

Of the 27 women who were invited to participate, 21 agreed and completed the telephone interview (mean age: 32.3 ± 3.0 years). Characteristics of those who completed the interview are summarized in Table 1, and the participant characteristics can be found in Table 2. There was no significant difference in weight change, BMI change, age or parity between those who did or did not agree to participate in the post-intervention interviews; however those who did not agree to an interview were significantly (*p* < 0.05) heavier at baseline (weight: 90.0 ± 9.2 kg vs. 77.5 ± 10.3 kg; *p* = 0.01) and at follow-up (90.6 ± 10.6 kg vs. 76.6 ± 10.6 kg; *p* < 0.01). The thematic network is presented in Figure 1 and is described in detail in the following sections.

### 3.1. Motivations and Expectations

#### 3.1.1. Motivations for Participating in VITAL Change for Mums

The participants were asked, “Tell me your reasons for wanting to take part in VITAL changes for mums.” Themes related to motivations included the following: to lose weight gained during pregnancy, motivation and accountability, inadequacy of current diet or physical activity, and to gain skills/knowledge.

Most participants were motivated to join VITAL to lose the excess weight gained during pregnancy. Many talked of having made previous weight-loss attempts, which for most had been only partially or not at all successful. Most talked of needing that extra bit of motivation to “get started” and hence volunteered to participate in VITAL:

*“I put on like over 30 kg when I was pregnant, so I was really keen for that and previously I used to enjoy exercising and in a gym or whatever it might be where I was finding it just really hard to find time so I it really appealed to me having the program that could be done in the home as well as including bub like going for walks and things like that.”*

*—Participant 17* (BMI: 30.3 kg/m^2^)

The participants voiced being motivated by an awareness of their own perceived inadequate diet and physical activity. There was an expectation that VITAL would provide the motivation, commitment and accountability to initiate and maintain such healthy lifestyle behaviours, which they found difficult to do “on their own”:

*“My biggest expectations I guess was having that accountability because it’s so easy to just be like ‘nahh’, so I really liked the accountability that someone was like saying ‘this is what you should be doing, have you done it’.”*

*—Participant 1* (BMI: 30.3 kg/m^2^)

#### 3.1.2. Expectations of VITAL Change for Mums

The participants were asked to describe how well their expectations were met, with the emergent themes being that VITAL was well-delivered and appropriate for mothers with young children and that participation developed a greater confidence in their ability to improve their own health-related behaviours. Many participants felt they had gained greater confidence in their ability to “take charge of their bodies” and had witnessed outcomes (body composition, weight and general health) that placed them at a different foundation from which they could bring about and sustain future lifestyle changes:

*“I thought it was a really positive experience and I was pleased with the outcome. I had some weight loss in the 8 weeks and even though I haven’t sustained the exercise as much as I would have liked, certainly some dietary changes have persisted and I feel like I’ve got at least a good base to move forward with some exercise modification once I get a bit of time.”*

*—Participant 4* (BMI: 22.6 kg/m^2^)

*“I just wanted to say thank you because I found, like it might sound weird, but I found it has been a life-changing program for me. I’ve been able to make some really positive changes with my eating and my exercise and I feel a lot better about that as a result of finishing the program.”*

*—Participant 13* (BMI: 32.5 kg/m^2^)

### 3.2. Participants’ Experience with the Mode of Delivery

The participants were asked “What was it like to participate in online consultations with these (dietitian and exercise physiologist) health professionals?” Themes included the perception that the video consultations did not differ to other consultations and were convenient to use and that the exercise physiology consultations had some logistical challenges.

Most participants said they did not feel that the video consultations had differed from a face-to-face experience, particularly in the case of the dietitian. It was indeed considered by all as a preferred alternative to standard health professional consultations as a result of the convenience and lack of logistical challenges commonly experienced by mothers of young children when attending appointments out of the home:

*“The reason I really liked it because I didn’t have to leave my house. So to get to like a half an hour appointment with my kids is a two hour two-and-a-half-hour expedition. And you know you have to pack snack, and activities for things to do, entertainment and they need it in that consultations when you can’t give your full attention so you would still look at your kids usually so I really like it for the fact that I could set the time of when they were resting or sleeping it was I could give my full attention so it was just really easy that way.”*

*—Participant 6* (BMI: 20.8 kg/m^2^)

A few participants commented on initially having been wary of this mode of delivery. However, any concerns and apprehensions held had been fully allayed, with many commenting on how “natural” the interaction had been:

*“You can still read body language and everything. I think, if anything, you’re a bit more comfortable because you’re at home and you're in a safe environment.”*

*—Participant 8* (BMI: 28.4 kg/m^2^)

The most frequently mentioned problem that the participants experienced in relation to the video consultations was that this mode of delivery inherently prevented the exercise physiologist from physically correcting movement, posture and technique, which was the only reason the participants had perceived the dietitian consultations to be more appropriate for this mode of delivery:

*“I think like comparing the dietitian and exercise physiologist, the dietitian was probably a bit easier to do via the video just ‘cause it was just sort of talking. The ones with the exercise physiologist I mean it was fine but I guess there was a couple of times she asked me to do certain exercises and it was a little bit hard to kind of like not be in person to get that across and to know exactly if I was doing the right thing, but overall yeah it worked quite well. … It was a good way to do it, especially being a mum and not always being able to get out of the house; so yep in that regard, it’s a really good option.”*

*—Participant 19* (BMI: 26.2 kg/m^2^)

#### 3.2.1. Dietitian Video Consultations

The participants were asked to describe the advice provided by the dietitian and what they thought about the goals set during video consultations. Themes that arose from their descriptions included the following: personable practitioner, appropriate for the mode of delivery, tailored and comprehensive content, realistic goals and gained knowledge and confidence.

The participants described their consultations with the dietitian very favourably. The dietitian was perceived as knowledgeable, motivating, encouraging and approachable, leading the participants to feel understood and listened to. The consultations were also perceived as appropriate for the mode of delivery and were delivered in a convenient, tailored manner, which was valued by many participants:

*“It was really tailored to my specific needs, talking about the kinds of foods I would eat and how often and how much, rather than just a template of you should do this and I just felt it was applied better to myself and I was able to talk openly to the dietitian about eating habits and stuff like that.”*

*—Participant 13* (BMI: 32.5 kg/m^2^)

Overall, the nutrition content was perceived to be comprehensive, realistic and individually tailored, rather than a one-fits-all approach. This advice had most often centred around making healthier (e.g., reducing saturated fat) choices, paying attention to portion sizes, and choosing healthier snacking options, with others having received guidance around changing the nutritional composition of their diet, or increasing fruit and vegetable intake:

*“It was good, like you know when she sort of reviewed my three day photo journal and gave me the heads up ... it was more my portion sizes than the actual foods I was eating; like I’m eating the right foods, I was probably eating a bit too much and the other thing that I found really helpful was she sent me through a link of different snack ideas because that’s probably my second weakness; like my main meals, I’m pretty good with but then it gets to snack time and I just grab whatever’s closest and so I found that really helpful so … I just stick it on the fridge and you know and then when I went shopping you know got some ideas and stuff like that so that was excellent for me.”*

*—Participant 7* (BMI: 28.1 kg/m^2^)

*“She listened well to what I’m saying; she looked at the food survey [Australian Eating Survey] and what I was eating throughout the course of the day and made sort of thoughtful, sensible adjustments, so it didn’t seem a one-size-fits-all program. And, yeah, it was evidence-based and science-based, which is important to me personally.”*

*—Participant 5* (BMI: 31.1 kg/m^2^)

All the participants had perceived the nutrition goals to be realistic, achievable and easy to implement and adopt into their existing routines. Most had viewed the goals as quite moderate and requiring of few drastic changes, which appeared to be important for adoption and sustainable integration into their everyday routine. Those who commented on this aspect had all felt the goals to be highly relevant to the needs identified and current circumstances. 

Some participants talked about the importance for themselves of the dietary consultations having been conducted in such a positive and empowering manner, feeling they instilled a sense of confidence in the ability to make effective and long-term changes.

Despite the previously stated positive comments, a few participants felt disappointed by the lack of specific diet plans, but acknowledged that this was more a problem relating to a clash with their expectations rather than an inherent weakness in the format of the program:

*“It’s different for everybody but I think if I was given ‘you must eat this to be healthy’, I probably would have followed that. Like as if you had an option of ‘do you want me to give you a diet plan for a week or whatever?’, I would have said ‘yes’ as opposed to, you know, change what you’re eating type thing. … I mean, it’s still worth a chat but … I think if there had been sort of like a dinner plan or something like that when I was feeling like quick healthy recipes, I would have followed that.”*

*—Participant 17* (BMI: 30.3 kg/m^2^)

Interestingly, a few participants alluded to feeling better equipped to deal with future weight gains/challenges (e.g., after further children) and a greater sense of knowledge and recognition of their individual challenges relating to healthy eating. Although the majority felt confident that they could maintain the changes made during VITAL, some participants reported that there were areas in which they had slipped back into old patterns and routines (e.g., full-fat options because of family preference, take-away because of being time-poor; changing routines because of being back at work, etc.):

*“(My diet is) not as good as it was during VITAL. Again, partly because I’ve gone back to work and I’m having to adjust and you know do my meal prep on Sunday and stuff like that so it’s just made life a bit more complicated I guess because my routine has changed. I think if I was still doing, you know, if I still wasn’t working it would have been ok.”*

*—Participant 11* (BMI: 31.3 kg/m^2^)

#### 3.2.2. Exercise Physiology Video Consultations

The participants were also asked to describe the advice provided by the exercise physiologist and what they thought about the goals set during video consultations. Themes that arose from their descriptions of these consultations included the following: motivating practitioner, tailored and flexible goals, and gained knowledge and confidence.

The participants were overall very favourable about their experiences with the exercise physiologist consultations. The exercise physiologist was perceived by all the participants to be very motivating, supportive and realistic, and offered advice that was highly flexible and tailored to needs, lifestyle and changing circumstances. The exercise physiologist had been perceived to work closely in conjunction with the participant to create a tailored and targeted program, provide advice that was aligned with personal goals, and most importantly, fit in with current routines, practices and capabilities. Particular mention was made of advice being tailored around existing barriers to exercise (e.g., time), current life circumstances (such as capitalising on current activities as part of their job), personal preferences and dislikes, current goals and fitness levels:

*“She gave me advice on strength-building exercises that I can do, which I had previously I hadn’t had a lot of familiarity with … and we talked about like the other types of exercise that I can do to increase my fitness during the week and things that fit in with my work routine and my home routine and talked about interval training, which I found was really beneficial and something that I hadn’t tried before. … I found she was able to help tailor a program specific to me and my needs, yeah, it was really great.”*

*—Participant 13* (BMI: 32.5 kg/m^2^)

*“I thought it was great. I’ve never seen an exercise physiologist before and I’ve seen personal trainers; I’ve had training programs written for me but I really really enjoyed it. It was very specific. It was targeted directly and tailored for me. … I was very impressed and I would probably, if I ever had problems doing exercise in the future, I would go to an exercise physiologist.”*

*—Participant 12* (BMI: 23.3 kg/m^2^)

All the participants felt positive about the exercise goals that were developed for them during the consultations with the exercise physiologist. The participants particularly put value on the fact that goals set had been specific and progressively challenging but achievable and sustainable. The participants described these as important features of their continued motivation, as their progressive sense of success imparted a sense of achievement and desire to continue and improve further:

*“Basically she set up a program for me, which I thought was really good, and it was well suited to my fitness levels so I felt that all the exercises that she gave me were appropriate and achievable. She was really thorough like in everything that she provided to me in details and plan, pictures and diagrams and everything; she also like demonstrated all of the exercises that she wanted me to do like the video call—she was really helpful.”*

*—Participant 19* (BMI: 26.2 kg/m^2^)

*“(The goals) were good because they were achievable but like motivated me and they so what has happened ahead of the program was its created a whole habit for me in terms of when I do my exercise and what I do for exercise time, which was something that I hadn’t been able to implement myself. I started to, but she really completed and gave me a whole weekly program I still could that was really good.”*

*—Participant 6* (BMI: 20.8 kg/m^2^)

For many, there appeared to be a greater recognition of the importance of becoming more active and that this necessarily involved a review of competing priorities in an often time-poor lifestyle. Certainly, motivation to make and maintain changes had improved for many, who felt more positive about their ability to be more active long-term despite various barriers:

*“I’ve been more motivated. I’ve just come back to work and I’ve sort of sat down and made sure I’ve made times and sort of planned into my schedule to fit some of this stuff in, which is all stuff we talked about. … I just felt, I don’t know, empowered the right word, but I felt like it was important to do that so and they gave me that motivation to keep it moving, which is good.”*

*—Participant 9* (BMI: 28.0 kg/m^2^)

#### 3.2.3. Number, Duration and Timing of Video Consultations

The participants were asked, *“Tell me your thoughts about the amount of contact you had with the dietitian/exercise physiologist.”* Emerging themes included the following: appropriate length of video consultations, flexible available times, and desire for increased frequency of contact.

For all the participants, the duration of the consultations was appropriate, and they had not felt rushed. The timing of the sessions was similarly perceived as appropriate by all, because of their flexibility. The participants had made a choice as to which professional to see for a third consultation on the basis mainly of their need for further information and guidance but also because of their perceived level of progress with regard to optimising diet and exercise habits. Of those participants completing an interview, seven chose to have an additional consultation with the exercise physiologist and five chose to with the dietitian. Some participants requested the third session with the exercise physiologist to sustain a sense of accountability to maintain motivation and further progression, while others had a desire to extend themselves further, or had perceived a need for further adjustments to be made to their goals or exercise program:

*“I’d be almost tempted to say maybe one a week would be really good. Only because I find if I have to answer to someone I think I will, you know, I’ve got to talk to [the exercise physiologist] on Friday so I better get my exercises done. You know what I mean? … But in saying that I made so much progress and got plenty out of it with the three that I had.”*

*—Participant 3* (BMI: 29.2 kg/m^2^)

Those who had not wished to have a third consultation with the exercise physiologist had either felt that their dietary habits required more attention, or more commonly, that they had perceived that their progress with their “prescribed activities” had not been sufficient to warrant further contact. Similarly, one participant noted a lack of “progress” or behavioural change regarding their dietitian consultations as a reason for not choosing a third:

*“I thought I was going to choose the third because I knew eating was my biggest problem and exercise was not a problem and easier for me to do, but in the end I thought, you know, with the dietitian I know what I should be doing, I’m just not doing it. So it was more like ‘ok, she’s given me these things that I need to remember, I know how to do it I just need to remember it’. So I’m not going to get on the phone and have her tell me the stuff again that I need to remember, if that makes sense.”*

*—Participant 12* (BMI: 23.3 kg/m^2^)

Conversely, some participants felt that the frequency of the consultations was not enough, reporting that their motivation declined toward the end of the program and that the implementation of more regular contact may have helped. A few commented that, in retrospect, they would have benefitted from another session:

*“I was going really well and she sort of said to me at the second consult you know there’s nothing else that you can do, just keep going. In hindsight, because my eating has probably fallen off the track a little bit, I should probably have had a third one, but by the time I sort of thought of it, you know, it was right at the end of the study so I didn’t worry about it. I think like the video consults were fairly early on in the piece; like it would have been nicer to have like a set session towards the end ‘cause I think that would be when people tend to fall of the wagon. Like, I feel most people are pretty gun-ho and keen and something but so I know that there was an option to have a third one, but because it wasn’t locked in and I just sort of had the choice to have it or not and I didn’t, I think I probably would have benefitted just to help keep me motivated and keep me going.”*

*—Participant 11* (BMI: 31.3 kg/m^2^)

*“And again it was for that same reason I said that maybe a later video consultation would have been good to keep you motivated and if you hadn’t been going so well it’s like, ‘ok, I’ve got to get back on track now and I’ve got time to make positive changes before the end of the study and on goal’.*

*—Participant 18* (BMI: 35.8 kg/m^2^)

### 3.3. Perceptions of the Components of VITAL 

The participants were asked, *“Tell me what you thought about the different program components?”* Themes relating to program components included the following: acceptable, easy to use, and minor technical issues.

#### 3.3.1. iPad Mini

Overall, the iPad was well received as the main tool for interaction and engagement with the program. The majority of the participants perceived the iPad to be easy and efficient to use. Most felt it was a good portable size, which facilitated the everyday interaction with the program. A few participants commented on minor problems. Two participants had resorted to downloading the program apps on their mobile phones instead as this was felt to be more portable and accessible to them. A few felt that the iPad was an awkward size (too small to be useful to view instructional pictures and type comfortably on, while too large to be convenient to carry around), or experienced problems holding/manipulating it during exercise physiology consultations. 

#### 3.3.2. VSee

Half of the participants found the VSee program easy to use, providing a good quality connection, with no problems:

*“It was a good program. I didn’t have any technical difficulties with it, which was great. I’ve used other video conferencing apps before like Skype and Facetime and sometimes you can get some sound issues, and with VSee it worked much better.”*

*—Participant 13* (BMI: 32.5 kg/m^2^)

A few participants experienced minor problems/issues, such as Internet problems, pixelated images, delayed sound or other minor quality-related issues, whereas the remainder had some problems with log-in (required re-login to make it work).

#### 3.3.3. Evernote

Most people expressed very positive attitudes towards the inclusion of the Evernote app. For many, it had provided a valuable reference and reminder of the information talked about during the sessions. It was also perceived as a user-friendly, easy-to-access and efficient avenue for the provision of follow-up information, as a means of prompting, and by some, as a vehicle for sustaining motivation:

*“I liked Evernote—I thought it was great. I thought it was excellent for me to be able to get back to because if I didn’t have those notes, I would have forgotten everything that they were telling me, so I felt it was very, very good.”*

*—Participant 12* (BMI: 23.3 kg/m^2^)

#### 3.3.4. Jawbone UP

While a quarter of the participants preferred using their existing activity monitors, another quarter liked the Jawbone UP, with a few having bought one of their own since the study’s completion. However, half of the participants had experienced some problems or dislikes relating to the Jawbone UP, with the most frequently mentioned issues relating to the lack of a screen for checking step counts or battery charge, anticipated inaccuracy in measuring steps and sleep, and failure of the Jawbone UP to sync with an iPad.

#### 3.3.5. Gymstick

All the participants who reported having used the Gymstick (*n* = 19) had enjoyed it. They found it portable, convenient and easy to use. Of the participants who reported not having used it, or using it very little, this was mainly due to a preference to go to the gym or resistance training, with the Gymstick not being part of their personalised program.

#### 3.3.6. Australian Eating Survey Dietary Assessment Tool

The majority of the participants found reviewing the AES personalised nutrition report provided important new information about their dietary intake and behaviours, which for some had been a confronting activity, but also acted as a catalyst for change:

*“I think and especially with her reference back to the food survey (Australian Eating Survey) and her drawing on that and saying ‘you know, this is what you’re lacking’, and it was clear for example that I wasn’t eating enough greens and in my mind I always hear her telling me ‘you know you got this, you may need to increase your greens’; so I mean that certainly helped because in the back of my mind I’m like ‘oh my God, I’ve got the lowest score are my vegetables and I thought I was a healthy person’; so I think having that measureable and again specific survey, it actually means something; although the survey was hard to understand but with her talking about it to me and telling me how to read it or understand it or basically giving me the facts from that the survey was enough for me to go on my own, ‘I need to change things’.*

*—Participant 12* (BMI: 23.3 kg/m^2^)

*“I actually thought it was a real eye opener … having to go through the survey like ‘oh, actually I haven’t realised until I have to write things down that I’m having ice cream three or four times a week you know’, so I found that quite helpful and a bit of a wake-up (call).”*

*—Participant 5* (BMI: 31.1 kg/m^2^)

## 4. Discussion

The aim of the current paper is to provide an insight into postpartum women’s experiences of engaging with a dietitian and exercise physiologist through video consultations to tailor nutrition and exercise care as part of a personalised healthy lifestyle intervention. This qualitative evaluation identifies the capacity for remote healthcare delivery to support women to achieve a healthy weight through lifestyle modifications following childbirth. Video consultations were perceived as acceptable, convenient, easy to use, supportive and beneficial.

Whilst interventions targeting PPWR have been frequently reported, there has been limited investigation into delivery by approaches incorporating technology [4]. Additionally, research reporting patient experiences of allied health-delivered video consultations is limited [17]. A recent scoping review investigating the use of eHealth and information communication technologies in health-service delivery in Australia identified only one study involving dietetics; however this did not include the delivery of video consultations [18]. No study reported the inclusion of exercise physiology services. Whilst patient experiences of physiotherapy [19,20] and speech pathology [21] video consultations have been positive, little is known about patient experiences of dietetic and exercise physiology services. This study provides the first insights into postpartum women’s perspectives and experiences of receiving tailored, one-on-one nutrition and exercise care via real-time video consultations.

Our formative research into factors that influence healthy eating and physical activity in women following childbirth identified a number of barriers, including a lack of time, knowledge and support [8]. Women are also undergoing a number of unique physiological changes in the first postpartum year, including changes in haemodynamics, musculoskeletal adaptations, and breastfeeding status, as well as emotional changes [22]. Further, women reported being motivated to improve their weight status for a variety of reasons, including improving self-confidence, mood and health [8]. It is important to note that these factors differed by sociodemographic, weight and pregnancy characteristics, highlighting the importance of tailoring advice and support to the individual [8,23]. This current study highlights the importance of providing tailored advice to women following childbirth. The participants perceived the individualization of the program to their specific situation as a key positive attribute of their video-consultation experience. Women consistently commented on the value of setting tailored behavioural goals with advice on the basis of their current reported eating and physical activity habits. Women particularly reported the value in receiving feedback on their performance with discussed behaviours and by reviewing set goals; this was perceived to be supportive and created a sense of accountability. Practitioners and researchers need to consider the unique circumstances faced by postpartum women (i.e., physiological changes, barriers relating to family and caring responsibilities) when providing lifestyle recommendations.

The Institute of Medicine recommends all postpartum women be offered counselling services to support healthy nutrition and physical activity behaviours to promote the attainment of a healthy weight after childbirth [24]. Despite these recommendations, women commonly do not receive [25] or seek professional advice [8]. Conversely, many women report attempting to make nutrition and physical activity changes themselves, often with little success [8,25]. Support services need to be accessible to new mothers and need to manage the identified need for childcare, time efficiency and convenience [8,26,27,28]. Previous studies have reported that the need for childcare is an influencer of intervention engagement. Women who participated in a 9 month lifestyle intervention, which included 8 healthy eating classes, 10 physical activity classes and 6 telephone counselling sessions, in the first year after childbirth reported that arranging childcare or organising their children to bring them to the scheduled classes was a barrier to attendance [29]. Participation in Ostybe et al.’s study was low, with a mean of only 3.8 classes and 3.3 telephone calls completed. In addition to childcare, the need to travel to health services has been reported previously as a barrier to participation for postpartum women [30]. Healthcare delivered remotely via video consultations offers an alternative to in-person care, potentially enabling women to overcome these barriers [31]. This can be seen in the current study, in which women viewed the video consultations favourably, given the ability to be at home with their child, without the need arrange their children to attend with them or travel to a physical location. 

Women in this study consistently commented on the benefit of having the support of the health professionals in maintaining their motivation to make lifestyle changes. This was evidenced by the preliminary efficacy results from VITAL change for mums, in which statistically significant improvements in waist circumference, body composition, cardiorespiratory fitness, dietary intake and physical activity were seen from baseline to 8 weeks [32]. In this qualitative analysis, women felt that the support they received improved their confidence to manage future weight, nutrition and physical activity challenges. Providing a one-to-one health practitioner experience via video conferencing may help to maintain engagement in lifestyle modifications [33] and thereby improve health behaviour outcomes, whilst also overcoming time, transportation and childcare barriers. A recent study in which postpartum women received a personalized lifestyle intervention delivered solely via a smartphone app reported difficulties with engagement and adherence despite providing near-real-time feedback on logged health behaviours and access to health information. Interestingly, in the current study, some women reported they would have found some additional health information (i.e., recipes or daily meal plan) to complement their video consultations beneficial. A combined approach providing a personalised face-to-face virtual experience in addition to access to a Web-based platform providing appropriate health resources and information (i.e., meal preparation suggestions and exercise demonstration videos) should be further explored.

Regular and ongoing support from qualified health practitioners is recommended in the management of overweight and obesity [34]. Women in the current study commented on the benefit of regular support from both practitioners and how the flexible consultation options were beneficial and appreciated. Women commented that it was difficult to stay motivated in the latter stages of the 8 week intervention if they did not opt to utilise their fifth consultation or no longer had any available video-consultation sessions remaining. Given that the Clinical Practice Guidelines for the Management of Overweight and Obesity in Adults, Adolescents and Children in Australia recommend individuals above a healthy weight receive regular follow-up (initially fortnightly) with clinicians, with ongoing, long-term support to last a minimum of 12 months [34], future research should adopt a sustained support approach during the intervention period to facilitate enhanced engagement with prescribed lifestyle modifications. The current study demonstrates that video consultations may be a practical medium to provide the required regular and sustained support, given the ability to provide a regular one-to-one experience that is flexible in delivery and overcomes time and access barriers. 

Access to technology and Internet connectivity is increasing, with 86% of Australian households [13] using the Internet. Just as many adults own a smartphone [35], and households report utilising multiple devices to access the Internet at home [13]. Given the wide-reaching use and availability of technology and Internet connectivity, healthcare providers have an opportunity to digitally integrate the provision of information and service delivery into patient-care models. This study provides novel patient perspectives on the experience of receiving healthcare information instantaneously and digitally via a file-sharing application (i.e., Evernote), using tablets (i.e., iPad mini) to interact with health professionals and completing health-related surveys prior to consultations to support practitioner recommendations (i.e., AES FFQ). In particular, the participants in this study favourably commented on being provided with their own results from the online dietary assessment using the AES personalised report to support the recommendations made by the dietitian. The participants reported positive experiences with both practitioners; however reported some difficulties relating to the practical nature of the exercise physiology video consultations. Exercise demonstrations were occasionally difficult to interpret or perform themselves with the device set-up. To improve the user experience, participants should be provided with set-up instructions relating to the tablet or Web-camera position and the space required for an exercise-based consultation. In this study, the participants were provided with personalised exercise programs including still images of prescribed activities, which were shared via Evernote. A future improvement to assist with the interpretation of exercise prescription and techniques may be to provide access to a Web-based or smartphone portal with an exercise video catalogue.

### Strengths and Limitations

This study is the first to provide an insight into women’s perceptions and experiences of receiving nutrition and exercise care via video consultations following childbirth. The individual interviews offered women the opportunity to explain their experiences in an environment that was flexible, given the need to manage family-related responsibilities. There was an even distribution of women in the healthy weight, overweight and obese weight-status categories who participated in VITAL; however there was a lack of diversity in terms of education attainment, marital status and household income. Responses to interviews were therefore not stratified by sociodemographic characteristics because of low participant numbers in these subgroups. The sample participants recruited into this study were of a relatively high education level, and hence caution is needed when interpreting the results, as they cannot be generalized to all post-partum women. Future research in a larger, more diverse sample of women is required. This should employ a recruitment strategy that targets women from a range of strata of both education and socioeconomic status to ensure women from all sociodemographic backgrounds are recruited and external validity is optimized.

## 5. Conclusions

Following childbirth, it is recommended that postpartum women receive support to improve their nutrition and physical activity behaviours in order to assist them in achieving and maintaining a healthy weight. However, women face unique and significant barriers to accessing health professionals to improve these lifestyle factors during the postpartum period. Video consultations provide an alternative to the provision of in-person, one-one-one health professional support and may overcome stated barriers. In the current study, women perceived video consultations to be convenient and appropriate. The receipt of tailored advice from the dietitian and exercise physiologist via video consultations was acceptable and improved the participants’ motivation to improve nutrition and physical activity behaviours. Future research should provide set-up instructions for exercise-based consultations to improve the interpretation of the exercise demonstrations and also consider the addition of a Web portal for additional sources of health information (i.e., recipe and meal ideas and exercise videos). Given the demonstrated feasibility and acceptability of video consultations to deliver nutrition and exercise care for postpartum women, further research in a fully powered randomised controlled trial to examine efficacy is warranted.

## Figures and Tables

**Table 1:** Baseline characteristics of participants completing a post-intervention interview following Video-coaching to assist lifestyle (VITAL) change for mums ^.

Variable	Mean ± SD or % (*n*)
Age (years)	32.3 ± 3.0
Height (cm)	165.8 ± 3.3
Parity	1.6 ± 0.9
1 child	61.9 (13)
2 children	23.8 (5)
≥3 children	14.3 (3)
Time since birth (days)	215 ± 61.4
3–6 months	28.6 (6)
6–9 months	47.6 (10)
9–12 months	23.8 (5)
Body Mass Index (kg/m^2^)	28.1 ± 3.8
Healthy weight (18.5–24.9 kg/m^2^)	23.8 (5)
Overweight (25.0–29.9 kg/m^2^)	38.1 (8)
Obese (≥30 kg/m^2^)	38.1 (8)
Country of birth	
Australia	90.5 (19)
Other	9.5 (2)
Marital status	
Married/de facto	100 (21)
Employment status	
Student	9.5 (2)
Full-time paid employment	33.3 (7)
Part-time paid (<35 h per week)	19.1 (4)
Not currently in paid employment	28.6 (6)
Other form of paid employment	9.5 (2)
Highest education level	
Certificate/diploma	19.1 (4)
University degree or higher	80.9 (17)
Household income ($AU)	
$1000–$1249 per week	14.3 (3)
$1250–$1499 per week	4.8 (1)
$1500–$1999 per week	23.8 (5)
$2000 or more per week	47.6 (10)
Do not want to answer	9.5 (2)

^ At enrolment to VITAL change for mums.

**Table 2:** Individual participant characteristics of women completing a post-intervention interview following Video-coaching to assist lifestyle (VITAL) change for mums.

Participant	Parity	Time since Birth (months)	BMI at Baseline (kg/m^2^)	Weight Change Pre–Post (kg) ^
1	1	9–12	30.30	−3.40
2	2	9–12	29.70	3.50
3	1	3–6	29.20	−4.60
4	2	6–9	22.61	−2.10
5	1	3–6	31.07	−2.50
6	4	6–9	20.84	−0.05
7	2	6–9	28.05	0.65
8	1	3–6	28.43	0.60
9	3	6–9	28.00	−0.35
10	3	6–9	25.87	0.25
11	1	6–9	31.33	−1.30
12	1	3–6	23.28	−0.70
13	1	9–12	32.52	−2.90
14	1	3–6	23.88	−3.00
15	2	3–6	30.76	−1.30
16	2	9–12	31.65	1.05
17	1	6–9	30.25	−5.65
18	1	9–12	35.81	2.50
19	1	6–9	26.23	1.45
20	1	6–9	25.53	−1.30
21	1	6–9	23.84	−3.75

^ Weight change reported following completion of the 8 week VITAL change for mums intervention.

## Supplementary Materials:

The following are available online at www.mdpi.com/2227-9032/6/1/8/s1. S1: VITAL change for mums interview protocol.

Supplementary File 1

## References

[1] Hollis J.L., Crozier S.R., Inskip H.M., Cooper C., Godfrey K.M., Harvey N.C., Collins C.E., Robinson S.M. (2017). Modifiable risk factors of maternal postpartum weight retention: An analysis of their combined impact and potential opportunities for prevention. Int. J. Obes..

[2] Martin J.E., Hure A.J., Macdonald-Wicks L., Smith R., Collins C.E. (2014). Predictors of post-partum weight retention in a prospective longitudinal study. Matern. Chil. Nutr..

[3] Rooney B.L., Schauberger C.W. (2002). Excess pregnancy weight gain and long-term obesity: One decade later. Obstet. Gynecol..

[4] Vincze L., Rollo M., Hutchesson M., Hauck Y., Macdonald-Wicks L., Wood L., Callister R., Collins C.E. (2018). Interventions including a nutrition component aimed at managing gestational weight gain or postpartum weight retention: A systematic review. JBISRIR.

[5] Gilinsky A.S., Dale H., Robinson C., Hughes A.R., McInnes R., Lavallee D. (2015). Efficacy of physical activity interventions in post-natal populations: Systematic review, meta-analysis and content coding of behaviour change techniques. Health Psych. Rev..

[6] Neville C.E., McKinley M.C., Holmes V.A., Spence D., Woodside J.V. (2014). The effectiveness of weight management interventions in breastfeeding women—A systematic review and critical evaluation. Birth.

[7] Van der Pligt P., Willcox J., Hesketh K.D., Ball K., Wilkinson S., Crawford D., Campbell K. (2013). Systematic review of lifestyle interventions to limit postpartum weight retention: Implications for future opportunities to prevent maternal overweight and obesity following childbirth. Obes. Rev..

[8] Vincze L., Rollo M.E., Hutchesson M.J., Burrows T.L., MacDonald-Wicks L., Blumfield M., Collins C.E. (2017). A cross sectional study investigating weight management motivations, methods and perceived healthy eating and physical activity influences in women up to five years following childbirth. Midwifery.

[9] Elliott-Sale K.J., Barnett C.T., Sale C. (2014). Exercise interventions for weight management during pregnancy and up to 1 year postpartum among normal weight, overweight and obese women: A systematic review and meta-analysis. Br. J. Sports Med..

[10] Amorim Adegboye A.R., Linne Y.M. (2013). Diet or exercise, or both, for weight reduction in women after childbirth. Cochrane Database Syst. Rev..

[11] Montgomery K.S., Bushee T.D., Phillips J.D., Kirkpatrick T., Catledge C., Braveboy K., O’Rourke C., Patel N., Prophet M., Cooper A. (2011). Women’s challenges with postpartum weight loss. Matern. Child Health J..

[12] American Telemedicine Association About Telemedicine 2016. http://www.americantelemed.org/main/about/about-telemedicine/telemedicine-faqs.

[13] Australian Bureau of Statistics (2016). 8146.0—Household Use of Information Technology, Australia, 2014–2015.

[14] Tong A., Sainsbury P., Craig J. (2007). Consolidated criteria for reporting qualitative research (COREQ): A 32-item checklist for interviews and focus groups. Int. J. Qual. Health Care.

[15] Novick G. (2008). Is there a bias against telephone interviews in qualitative research?. Res. Nurs. Health..

[16] Sweet L. (2002). Telephone interviewing: Is it compatible with interpretive phenomenological research?. Contemp. Nurse.

[17] Kelly J.T., Reidlinger D.P., Hoffmann T.C., Campbell K.L. (2016). Telehealth methods to deliver dietary interventions in adults with chronic disease: A systematic review and meta-analysis. Am. J. Clin. Nutr..

[18] Iacono T., Stagg K., Pearce N., Hulme Chambers A. (2016). A scoping review of Australian allied health research in ehealth. BMC Health Ser. Res..

[19] Hinman R.S., Nelligan R.K., Bennell K.L., Delany C. (2017). “Sounds a bit crazy, but it was almost more personal”: A qualitative study of patient and clinician experiences of physical therapist-prescribed exercise for knee osteoarthritis via Skype™. Arthritis Care Res..

[20] Lawford B.J., Bennell K.L., Kasza J., Hinman R.S. (2017). Physical therapists’ perceptions of telephone- and internet video-mediated service models for exercise management of people with osteoarthritis. Arthritis Care Res..

[21] Burns C.L., Ward E.C., Hill A.J., Kularatna S., Byrnes J., Kenny L.M. (2017). Randomized controlled trial of a multisite speech pathology telepractice service providing swallowing and communication intervention to patients with head and neck cancer: Evaluation of service outcomes. Head Neck.

[22] Romano M., Cacciatore A., Giordano R., La Rosa B. (2010). Postpartum period: Three distinct but continuous phases. J. Prenatal Med..

[23] Satia J.A. (2009). Diet-related disparities: Understanding the problem and accelerating solutions. J. Am. Diet. Assoc..

[24] Institute of Medicine (US) (2009). Weight Gain during Pregnancy: Reexamining the Guidelines.

[25] Dinsdale S., Branch K., Cook L., Shucksmith J. (2016). “As soon as you’ve had the baby that’s it…” a qualitative study of 24 postnatal women on their experience of maternal obesity care pathways. BMC Public Health.

[26] Chang M.W., Nitzke S., Guilford E., Adair C.H., Hazard D.L. (2008). Motivators and barriers to healthful eating and physical activity among low-income overweight and obese mothers. J. Am. Diet. Assoc..

[27] Doran F., Davis K. (2011). Factors that influence physical activity for pregnant and postpartum women and implications for primary care. Aust. J. Prim. Health.

[28] Nicklas J.M., Zera C.A., Seely E.W., Abdul-Rahim Z.S., Rudloff N.D., Levkoff S.E. (2011). Identifying postpartum intervention approaches to prevent type 2 diabetes in women with a history of gestational diabetes. BMC Pregnancy Childbirth.

[29] Ostbye T., Krause K.M., Lovelady C.A., Morey M.C., Bastian L.A., Peterson B.L., Swamy G.K., Brouwer R.J., McBride C.M. (2009). Active mothers postpartum: A randomized controlled weight-loss intervention trial. Am. J. Prev. Med..

[30] Berry D., Verbiest S., Hall E.G., Dawson I., Norton D., Willis S., McDonald K., Stuebe A. (2015). A postpartum community-based weight management intervention designed for low-income women: Feasibility and initial efficacy testing. J. Natl. Black Nurses Assoc..

[31] Rollo M.E., Burrows T., Vincze L.J., Harvey J., Collins C.E., Hutchesson M.J. (2017). Cost evaluation of providing evidence-based dietetic services for weight management in adults: In-person versuseHealth delivery. Nutr. Diet..

[32] Vincze L., Rollo E.M., Hutchesson M., Callister R., Collins C. (2018). VITAL change for mums: a feasibility study investigating tailored nutrition and exercise care delivered by video-consultations for women 3–12 months postpartum. J. Hum. Nutr. Diet..

[33] Rollo M.E., Hutchesson M.J., Burrows T.L., Krukowski R.A., Harvey J.R., Hoggle L.B., Collins C.E. (2015). Video consultations and virtual nutrition care for weight management. J. Acad. Nutr. Diet..

[34] National Health and Medical Research Council (2013). Clinical Practice Guidelines for the Management of Overweight and Obesity in Adults, Adolexcents and Children in Australia.

[35] Deloitte (2016). Mobile Consumer Survey 2016. http://landing.deloitte.com.au/rs/761-IBL-328/images/tmt-mobile-consumer-2016-final-report-101116.pdf.

