# Supplementary material for: Postpartum Women’s Perspectives of Engaging with a Dietitian and Exercise Physiologist via Video Consultations for Weight Management: A Qualitative Evaluation
Source: Healthcare (Basel). 2018 Jan 19;6(1):8. doi: 10.3390/healthcare6010008 (PMC5872215; doi:10.3390/healthcare6010008)
Supplement: Supplementary File 1 [file healthcare-06-00008-s001.pdf]

S1: VITAL change for mums interview protocol

Interviewer: Record start time

Introduction

Firstly, thank you for your participation in VITAL change for mums and also for taking the time to complete this interview with me today. We will be asking you a range of questions regarding the different aspects of your participation in the program. No answer is right or wrong; we value your honest opinion. Provide as much detail as you feel comfortable with. We will be covering the following topics: your expectations, your video-consultations experience overall, the Dietitian and Exercise Physiology consultations separately and the technology and equipment. The interview is expected to take 20-40 minutes.

Part One: Expectations and interest in participation

1. Tell me your reasons for wanting to take part in VITAL change for mums?
   Probe: What did you initially expect from VITAL change for mums?

2. On a scale of 1 to 5 (1 being the lowest rating and 5 being the highest rating), how well were your expectations met?
   Probe: Can you explain your reasons for selecting this?

Part Two: Video-consultation experience

As a part of VITAL change for mums you participated in online video-consultations with a Dietitian and Exercise physiologist.

3. What was it like to participate in online consultations with these health professionals?
   Probe to identify likes, dislikes, suggested changes?
   Probe to compare this experience to consulting a health professional (e.g. GP, dietitian, Ex Phys) face-to-face?

Part Three: Advice (Dietitian)

During VITAL for mums, you received a minimum of 2 video-consultations with the Dietitian. During these consultations you set goals to change nutrition and eating behaviours.

4. Tell me about the advice you received?
   Probe to identify the degree to which the Dietitian understood their needs, goals, barriers, tailored the advice based on their needs and expectations?
   Probe to expand / clarify response by probing on motivation, encouragement, Dietitian preparation, knowledge level, skill, etc

5. What did you think about the nutrition goals you set during your video-consultations?
   Prompts: Were they helpful, relevant, appropriate.

Following your video-consultations, the Dietitian used Evernote to provide you with your session summary and any resources (e.g. snack suggestions) discussed in the session.

6. Tell me your thoughts about the information received in Evernote?
   Prompts: Was it helpful, convenient to access.

7. On a scale of 1 to 5 (1 being the lowest rating and 5 being the highest rating), how well did the consultations with the Dietitian meet your expectations?
   Probe: Can you explain your reasons for selecting this?

8. Tell me your thoughts about the amount of contact you had with the Dietitian?
   Prompt: Frequency - how many consultations and reasons for (not) choosing a 3rd
   Duration - 60 minute initial and 30 minute review
   Timing - what time of day the consultations were available

9. Since being involved in VITAL, what have your nutrition and eating behaviours been like?
   Prompt: If your eating habits haven't changed, what are reasons they didn't change?
   Prompt: What plans, if any, do you have to maintain any changes?

Part Four: Advice (Exercise Physiologist)

During VITAL for mums, you received a minimum of 2 video-consultations with the Exercise Physiologist. During these consultations you set goals to change exercise and physical activity behaviours.

10. Tell me about the advice you received?
    Probe to identify the degree to which the Exercise Physiologist understood their needs, goals, barriers, tailored the advice based on their needs and expectations?
    Probe to expand / clarify response by probing on motivation, encouragement, Exercise Physiologist preparation, knowledge level, skill, etc

11. What did you think about the exercise goals you set during your video-consultations?
    Prompts: Were they helpful, relevant, appropriate.

Following your video-consultations, the Exercise Physiologist used Evernote to provide you with your session summary and any resources (e.g. exercise plans) discussed in the session.

12. Tell me your thoughts about the information received in Evernote?
    Prompts: Was it helpful, convenient to access.

13. On a scale of 1 to 5 (1 being the lowest rating and 5 being the highest rating), how well did the consultations with the Exercise Physiologist meet your expectations?
    Probe: Can you explain your reasons for selecting this?

14. Tell me your thoughts about the amount of contact you had with the Exercise Physiologist?
    Prompt: Frequency - how many consultations and reasons for (not) choosing a 3rd
    Duration - 60 minute initial and 30 minute review
    Timing - what time of day the consultations were available

15. Since being involved in VITAL, what have your exercise behaviours been like?
    Prompt: If your physical activity behaviours haven't changed, what are reasons they didn't change?
    Prompt: What plans, if any, do you have to maintain any changes?

Part Five: Technology and Equipment

VITAL change for mums provided you with an iPad mini for consultations. You conducted the consultations through VSee on the iPad. Evernote was the file sharing app where the Dietitian and Exercise Physiologist wrote your session summaries and shared resources. You also uploaded your 3-day photographic food record here. You were provided with a Jawbone to track your step count and a Gymstick for resistance based exercise. You also received your Australian Eating Survey report after your first Dietitian video-consultation.

16. Tell me what you thought about the different program components
    Prompt: iPad, VSee, Evernote, Jawbone and UP app, Gymstick, AES report, food record
    Probe to identify problems, what they liked, what they would change, would they keep using the Gymstick and Fitness tracker (Jawbone)

Part Six: Other

17. Before we end today, is there anything else you would like to tell me about VITAL change for mums?

Closing Statement

That concludes the interview today. Again, thank you for your participation in VITAL change for mums, and for taking the time to speak with me today. Should you have any further questions about the research you can contact Lisa any time. Take care and good-bye.

Interviewer: Record end time
